# Salivary AQP9 mRNA expression is associated with caries and periodontitis prevalence

**DOI:** 10.1038/s41598-026-37980-3

**Published:** 2026-02-13

**Authors:** Markus Baumann, Katharina Rump, Dominik Ziehe, Bjoern Koos, Michael Adamzik, Jennifer Orlowski, Martin Kunkel, Daria Pakosch-Nowak

**Affiliations:** 1 Zahnarztpraxis Sprockhövel, Dr. Markus Baumann, M.Sc., M.Sc., M.Sc, Sprockhövel, Germany; 2 Ruhr University Bochum, Knappschaft Kliniken University Hospital Bochum, Department of Anesthesiology, Intensive Care Medicine and Pain Therapy, Bochum, Germany; 3 Ruhr University Bochum, Department of Oral and Maxillofacial Surgery, Knappschaft Kliniken University Hospital Bochum, Bochum, Germany

**Keywords:** AQP9, Saliva mRNA expression, Caries, Periodontitis, Biomarker, Biomarkers, Dentistry

## Abstract

Dental caries and periodontitis are the most frequently diagnosed dental diseases worldwide. Their occurrence is age-dependent and, due to their widespread prevalence, they represent a major public health challenge. Further research is essential to identify factors that can be used to develop more effective prevention strategies. Potential factors contributing to the development of caries and periodontitis may be identified at the molecular level. Aquaporins (AQPs) are interesting candidate molecules in this context. Among them, AQP9, known for its role as a permeable channel for water, glycerol and other small molecules, has attracted considerable scientific interest in recent years. In this study we examined if AQP9 could serve as a biomarker for caries and periodontitis. The OKAPI study, a prospective observational study, enrolled patients in a dental practice and an oral and maxillofacial plastic surgery clinic. Clinical data like PSI-index and DMFT-Index were recorded and documented. Saliva samples were collected and RNA was isolated from saliva. cDNA was synthesized from the samples and qRT-PCR was performed using specific primers for AQP9 and ACTB (*n* = 159; 40.9% male). 39.4% of probands had severe caries and 15.3% had periodontitis. AQP9 mRNA expression was increased in patients with severe caries compared to milder forms (*p* = 0.0120), and increased AQP9 expression was also detected in patients with periodontitis compared to unaffected individuals (*p* = 0.0364). An AQP9 cut-off value was calculated to discriminate patients with and without severe caries. The groups below and above the cut-off differed only in the variables presence of severe caries or periodontitis and age: a binary logistic regression model was used to identify risk factors for caries (*p* = 0.009) and periodontitis (*p* = 0.023) and led to the conclusion that the AQP9 cut-off was most relevant. AQP9 mRNA expression is associated with the prevalence of caries or periodontitis and could represent a novel biomarker.

**Supplementary Information:**

The online version contains supplementary material available at 10.1038/s41598-026-37980-3.

## Introduction

Dental caries and periodontitis are the most frequently diagnosed dental diseases in Germany^1^. Their occurrence is age-dependent, and due to their widespread prevalence, they pose a significant public health challenge. Dental caries, in particular, is a global issue, affecting 2.5 billion individuals with untreated cavities in permanent teeth as of 2015^2^. The prevalence of dental caries in permanent teeth reaches its highest-level during adolescence and typically stabilizes afterward. Consequently, preventing caries during adolescence is crucial for maintaining optimal oral health throughout life^3^. Periodontitis affects a substantial portion of the population. In 2014, the prevalence of severe periodontitis was 10.4% among individuals aged 35–44 years and increased to 24.6% among those aged 65–74 years^4^. Current strategies for preventing caries and periodontitis focus on diet and oral hygiene practices; however, these measures are insufficient for fully preventing them in certain risk- populations^4,5^ and new contributing factors should be identified at molecular level. Due to its non-invasive accessibility and continuous interaction with oral tissues, saliva represents a promising medium for biomarker discovery, with numerous studies demonstrating its potential to reflect both oral and systemic disease states through the detection of DNA, RNA, proteins, and metabolites^6^. Aquaporins (AQPs) represent interesting candidate molecules in this context. AQPs are a family of membrane proteins that play a central role in water and glycerol metabolism across various tissues^7^. Our recent findings revealed that AQP9 mRNA is the second most prevalent aquaporin mRNA in human saliva, surpassed only by AQP5^8^. AQP9 is known for its role as a permeable channel for water, glycerol, and other small molecules, has gained significant scientific interest in recent years^9^. While AQP9 has been primarily studied in liver cells, macrophages, and certain brain cells, emerging research suggests it may also have a function in saliva^10^.

Studies on AQP9 expression in salivary gland tissue and their function in the oral cavity are still scarce. Initial indications of its potential function come from investigations into immune defense and metabolism in the oral cavity^9^. Given that AQP9 is expressed in immune cells, it could play a role in regulating inflammatory processes in saliva^11^. One of the potential roles of AQP9 in saliva lies in its involvement in caries formation. Dental caries is primarily driven by the interaction between oral microbiota, dietary sugars, and host factors, including saliva composition^12^. Investigating AQP9 in saliva could have far-reaching clinical applications. Additionally, modulating AQP9 could be a therapeutic option for improving oral health or treating inflammatory conditions in the oral cavity. Although the role of AQP9 in saliva remains largely unexplored, current evidence suggests it represents an important area of research. AQP9’s unique permeability for water and glycerol, as well as its involvement in immune processes, could have significant implications for oral health and systemic diseases. In this study we analyzed the AQP9 mRNA level in saliva of dental patients and tested if it is correlated to periodontitis or caries. Accordingly, this study addresses the question of whether AQP9 mRNA expression in saliva differs between individuals with and without caries and/or periodontitis, thereby evaluating its suitability as a potential biomarker for these diseases.

## Methods

### Study design and cohort

The OKAPI study (German Clinical Trial Registry No. DRKS00032425, date of registration: 16.08.2023) prospectively enrolled patients fulfilling the inclusion criteria in a dual centric approach. The study was approved by the Ethics Committee of the medical faculty of the Ruhr University Bochum (23-7821-BR, date of approval: 07.06.2023) and the Ethics committee of the Westphalia-Lippe Medical Association (2023-416-b-S date of approval 26.07.2023). The protocol, site-specific informed consent forms, participant education and recruitment materials, and other requested documents—and any subsequent modifications—also were reviewed and approved by the ethical review bodies. This study was conducted in accordance with the revised Declaration of Helsinki, good clinical practice guidelines, and local regulatory requirements. Patients (*n* = 325) were recruited after written informed consent over a period of 17 months at a dental practice. Eligible for inclusion were adult dental patients aged 18–75 years. The exclusion criteria were: genetically determined structural disorders of the dental hard tissue (e.g. amelogenesis imperfecta, dentinogenesis imperfecta, odontogenesis imperfecta), dementia and/or psychotic illness, lack of capacity to consent, insufficient knowledge of the German language to understand the scope and participation in the study.

### Clinical data

#### Dental caries definition

Dental caries experience was assessed using the Decayed, Missing, and Filled Teeth (DMFT) index, which quantifies the cumulative number of teeth that are decayed (D), missing due to caries (M), or filled (F). The maximum achievable DMFT score is 28, as third molars (wisdom teeth) were not included in the assessment. When assessing the prevalence of caries, a patient is considered to have caries if at least one of the following criteria is met:

- Active dental caries on one or more teeth
- Missing tooth/teeth (extraction due to caries)
- Presence of one or more restored teeth by means of a filling or prosthetic restoration (e.g. dental crown).

Due to the absence of a standardized WHO definition for caries severity, we established cohort-specific categories based on statistical distribution. Given a median caries involvement of 12 teeth, severity was classified as mild (< 9 teeth), medium (10–14 teeth), and severe (≥ 15 teeth).

#### Periodontitis definition

Periodontitis was defined as follows: In order to assess the gingiva the dentition is divided into six different sections (= sextants [S1-6]). These sections include the following teeth according to the Fédération Dentaire Internationale- tooth numbering system: The Fédération Dentaire Internationale (FDI) system, also known as the ISO 3950 system, is a globally accepted method for identifying teeth, using a two-digit code in which the first digit denotes the quadrant (1–4 for permanent teeth; 5–8 for primary teeth) and the second digit indicates the tooth position within that quadrant, numbered from the midline (1 = central incisor) to the third molar (8). In this study, the dentition was divided into six sextants (S1–S6) as follows:

- S1: 18–14 (upper right posterior)
- S2: 13–23 (upper anterior)
- S3: 24–28 (upper left posterior)
- S4: 38–34 (lower left posterior)
- S5: 33–43 (lower anterior)
- S6: 44–48 (lower right posterior)

Each section is examined with a periodontal probe. The highest probing depth of the respective sextant results in the respective Periodontal Screening Index (PSI) Index:

- Code 0: probing depth < 3.5 mm, no bleeding on probing, no calculus, no protruding filling/crown margins
- Code 1: probing depth < 3.5 mm, bleeding on probing, no calculus, no protruding filling/crown margins
- Code 2: probing depth < 3.5 mm, calculus and/or protruding filling/crown margins
- Code 3: probing depth 3.5–5.5 mm
- Code 4: probing depth > 5.5 mm

The highest PSI index of all six sextants gives the overall result (0–4), which results in the following interpretation:

- Total code 0: healthy
- Total code 1 or 2: gingivitis
- Total code 3 or 4: periodontitis

In addition to recording the caries and periodontitis status and collecting biosamples clinical data and risk profiles were collected by a questionnaire. This survey includes the following measurement variables:

- Age
- Gender (m/f/d)
- Pre-existing conditions: arterial hypertension, [non-]insulin-dependent diabetes mellitus
- Cardiovascular diseases [including CHD, post-myocardial infarction, PAD, etc.]
- Nicotine abuse
- Current or previous malignant tumor disease: localization, TNM stage, radio chemotherapy
- Long-term medication: bisphosphonates

### Collection of samples

As part of the dental treatment in the dental practice, patients included in the study, an oral mucosal swab and 2 ml saliva samples were obtained. All saliva samples were collected before dental treatment and after a fasting period of at least 30 min. RNA-preserving collection tubes (Norgen Biotek, Thorold, ON, Canada, disturbed by BioCAT, Heidelberg, Germany)) were used, and RNA integrity was assessed by spectrophotometry (Nanodrop, Thermo Fisher, Waltham, Massachusetts, USA). Samples were processed and stored at − 80 °C to ensure consistent quality. Samples with low quality were excluded from analysis (supplementary Fig. 1). The saliva samples were used to isolate RNA with the Total RNA Purification Kit (Norgen Biotek, Thorold, ON, Canada, disturbed by BioCAT, Heidelberg, Germany). After the isolation, the samples were stored at − 80 °C.

### RNA quantification

RNA samples of 135 patients were included in the RNA quantification analysis. 1 µg RNA was utilized for cDNA synthesis using high-Capacity cDNA Reverse Transcription Kit (Thermo Fisher Scientific, Wilmington, USA). For the expression analysis of interesting candidate genes, the RNA was transcribed into cDNA (cDNA reverse transcription Kit, Thermo Fisher, Wilmington, USA) and analyzed using qPCR. Specific primers (MWG eurofins, Ebersberg, Germany) for AQP9: AQP9_RT_Se: 5′-ATGTGGGAGCCCAGTTCTTG-3′; AQP9_Rt-As: 5′-TACGGAGCTGGGTATGTTGC-3′ were utilized and the expression was quantified by using the ΔCt-Method, by the usage of reference gene β-Actin (ACTB) as described earlier^13,14^.

### Statistical analysis

Patients characteristics are reported as percentages for categorical variables and as means with SD or medians with interquartile ranges (25th and 75th percentile), as appropriate. Categorical variables were compared using McNemar or Fisher’s exact tests. Continuous independent variables were compared using the Student’s t-test or the Mann–Whitney U test after testing for normal distribution using Shapiro–Wilk Test. The sample size calculation was performed using G*Power 3.1 (Heinrich Heine University Düsseldorf, Germany). Assuming a moderate effect size (Cohen’s d = 0.5), a two-tailed α = 0.05, and a power of 0.80, a minimum of 64 participants per group (128 in total) was required to detect statistically significant differences in AQP9 mRNA expression between groups (e.g., with vs. without caries). To account for possible data loss and unequal distribution of the subgroups, we included 160 patients in the final study cohort, where 24 patients had to be excluded (supplementary Fig. 1).

To determine the optimal cut-off value of AQP9 mRNA expression for distinguishing between patients with and without severe caries, and between patients with and without periodontitis, Receiver Operating Characteristic (ROC) curve analysis was performed. For each ROC curve, the Youden Index (J = Sensitivity + Specificity − 1) was calculated to identify the threshold with the highest combined sensitivity and specificity, representing the point of maximal diagnostic accuracy.

The resulting cut-off values were subsequently used in binary logistic regression models to evaluate their association with disease occurrence. These models were adjusted for potential confounding variables, including age, smoking status, and comorbid conditions such as hypertension, cardiovascular disease, and diabetes. To avoid overfitting, the correlation between the variables as determined using Spearman-Correlation analysis. The model was conducted without an interaction term to examine the independent effects of age and cut-off value on disease likelihood.

For each ROC analysis, the area under the curve (AUC) along with 95% confidence intervals (CI) were reported. Statistical significance was assessed using a *p*-value based on the null hypothesis that AUC = 0.5, indicating no discriminatory power.

The *p*-values for age and cut-off value were calculated to determine the significance of their effects. A probability curve was generated for different age values, predicting the likelihood of disease for values above and below the cut-off.

A line plot was created to visualize the relationship between age and disease probability, with a threshold line at 50% probability. The *p*-values for both age and cut-off value were reported in the plot title to indicate the significance of these variables.

Due to the single-cohort design, no external validation was performed; however, the robustness of the identified AQP9 cut-off values was supported by subsequent logistic regression analyses demonstrating their independent association with disease prevalence. Future validation in an independent cohort is warranted.

A *p*-value of lower than 5% was considered significant. If not stated otherwise, the data is always depicted as mean ± standard deviation (SD). All analyses were performed using SPSS (version 28, IBM, Chicago, IL, USA). GraphPad Prism 9 (Graph-Pad, San Diego, CA, USA) was used for graphical presentations.

## Results

In this study saliva samples from 135 patients were finally analyzed. The mean age of the cohort was 47 years and 40.9% were male (Table 1). 39.4% of the patients had severe caries and 15.3% had periodontitis (Table 1).

- 1. AQP9 expression is increased in patients with severe caries and periodontitis

**Table 1:** Baseline characteristics of the study cohort.

Characteristics (*n* = 135)	Mean ± SD	Minimum to maximum
Year of birth	1977 ± 14	1946–2003
Age	47 ± 14	21–77
Nicotine consume in years	5.5 ± 11.5	0–50
Gender male	56 (40.9%)	
Arterial hypertonia	23 (17%)	
Cardiac disease	11 (8%)	
Smoker	37 (27%)	
Diabetes	6 (4.4%)	
Cancer Bladder Mamma	3 (2.2%)	
Mild caries	30 (21.9%)	
Medium caries	52 (38%)	
Severe caries	54 (39.4%)	
Periodontitis	21 (15.3%)	

**Fig. 1:**
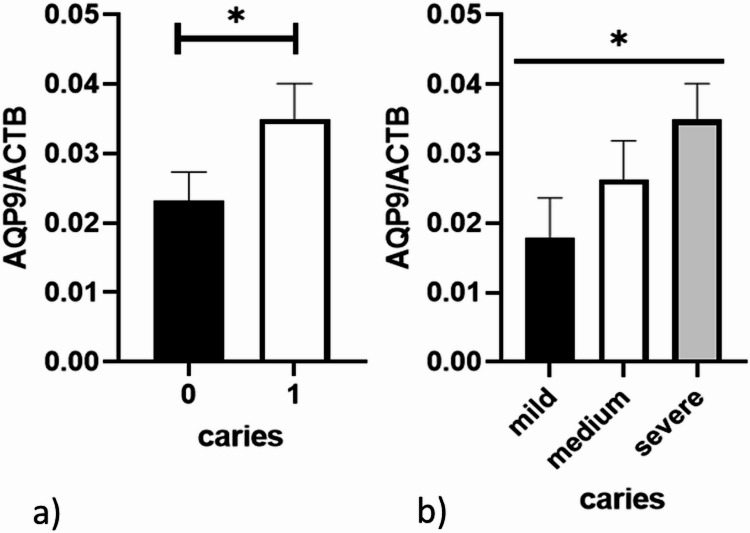
AQP9 expression in saliva samples of dental patients stratified by the occurrence of **a** severe caries (*n* = 135), or **b** three types of caries (*n* = 135).

**Fig. 2:**
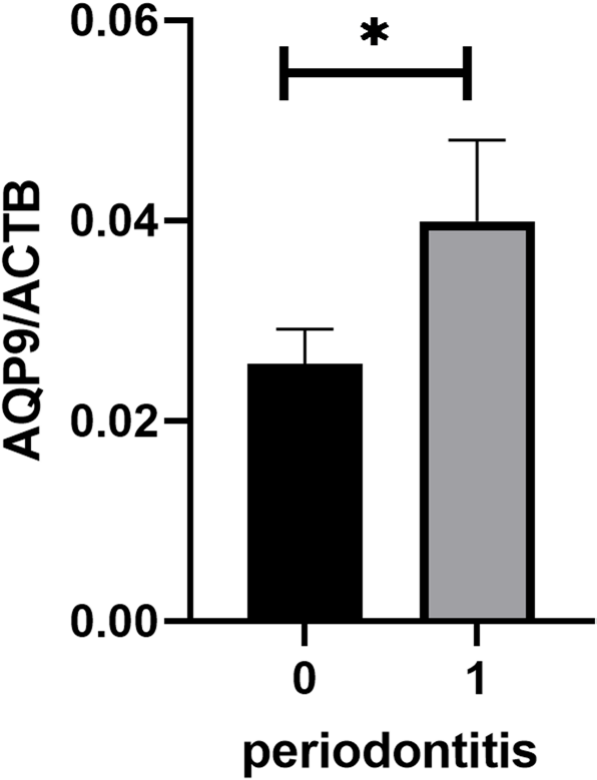
AQP9 expression in saliva samples of dental patients stratified by the occurrence of periodontitis (*n* = 135).

In a first step AQP9 mRNA expression was measured in saliva samples. AQP9 mRNA expression was increased in patients with severe caries compared to milder forms (*p* = 0.0120, Fig. 1a) and showed an increase from mild to moderate to severe forms (*p* = 0.0434, Fig. 1b). In addition, an increase of AQP9 expression could also be detected in patients with periodontitis compared to not affected persons (*p* = 0.0364, Fig. 2).

- 2. Determining of a cut off value for caries prediction

**Fig. 3:**
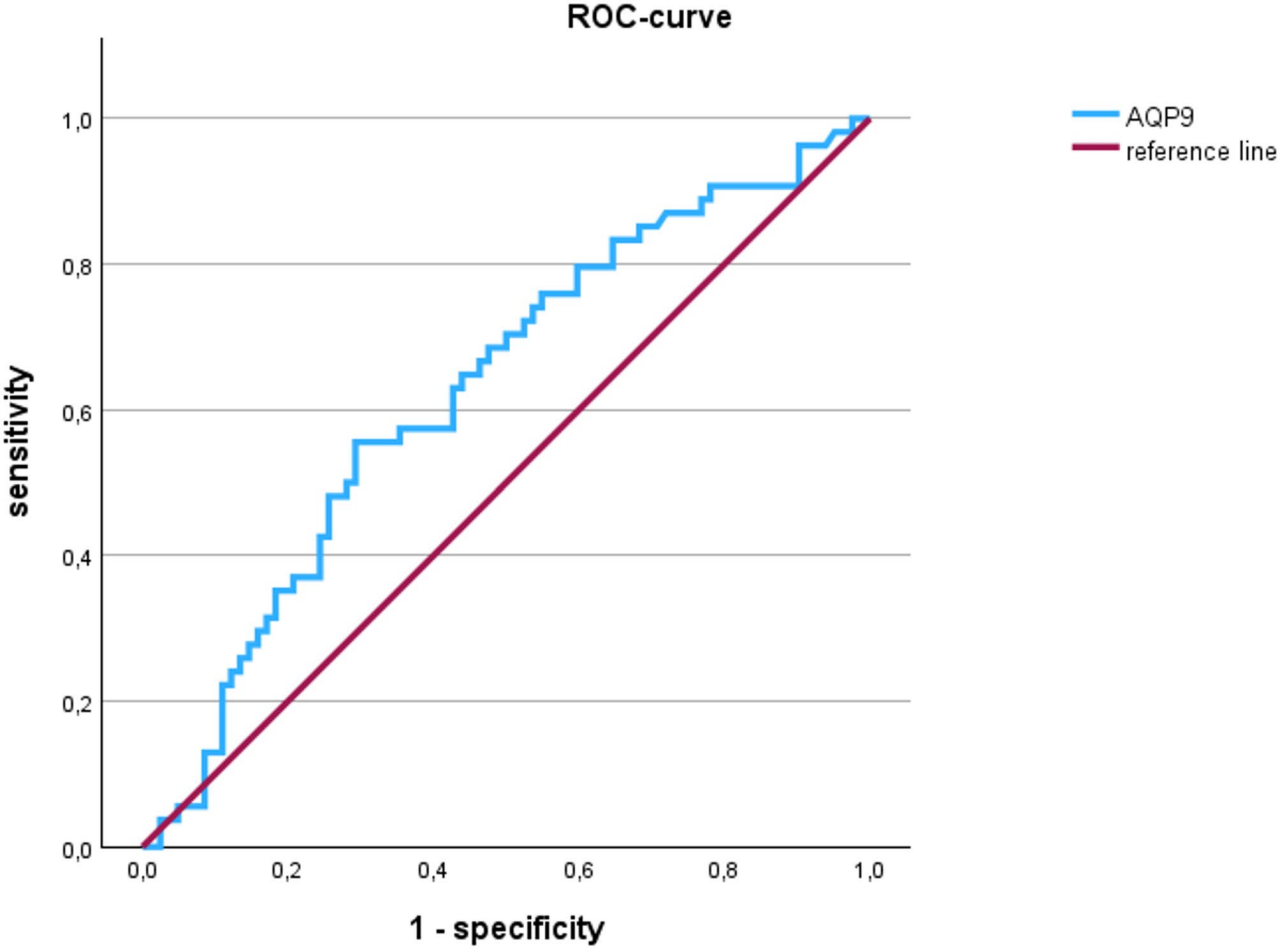
Receiver operating characteristic curve performed for AQP9 mRNA expression and the occurrence of severe caries.

ROC analysis was performed to find the point of best discrimination between severe caries and non-severe caries and between periodontitis and no periodontitis. The AUC for caries prediction was 0.627 (95% CI 0.531–0.723, *p* = 0.009, Fig. 3) and for periodontitis prediction 0.643 (95%CI: 0.521–0.766, *p* = 0.021). With Youden index cut-off values were calculated and were 0.0162 for periodontitis and 0.02096 for caries.

Cross tables were used to detect differences in the patient group between AQP9 below and above cut-off for caries. Due to the low number of periodontitis cases no cross tables were applied here. The groups below and above the cut-off differed only in the variables presence of severe caries or periodontitis and age (*p* < 0.05, Table 2).

**Table 2:** Comparison in the baseline parameters between patients in group above and below cut-off using cross-tables.

	Below cut-off	Above cut-off	*p*-value
Smoking (yes)	18 (22%)	19 (35.2%)	0.090
Arterial hypertonia (yes)	15 (18.5%)	8 (14.8%)	0.376
Diabetes	0 (0%)	1 (1.9%)	0.397
Cancer	0	3 (5.6%)	0.06
Severe caries	24 (29.3%)	30 (55.6%)	0.002
Periodontitis	7 (8.5%)	14 (25.9%)	*0.007*
Age < 35	25 (30.5%)	7 (13%)	*0.008*
Age 35–50	28 (34.1%)	14 (25.9%)	
Age > 50	29 (35.4%)	33 (61.1%)	
Gender male	32 (38.6%)	24 (44.4%)	0.591

The groups were compared by Chi-square test.

Significant values are in [italics].

In order to perform logistic regression analysis, the correlation of different variables was analyzed to avoid overfitting. The cut-off values for AQP9 caries or periodontitis were positively correlated with age (r = 0.199; *p* = 0.020 for AQP9 cut-off periodontitis; r = 0.257; *p* = 0.002 cut-off caries) cancer (r = 0.172; *p* = 0.046 for AQP9 cut-off periodontitis; r = 0.158; *p* = 0.031 cut-off caries) and to each other (r = 0.927; *p* < 0.001). Hence these variables were excluded from the regression model. In addition, the occurrences of caries (r = 0.472; *p* < 0.001) and periodontitis (r = 0.234; *p* < 0.001) were strongly correlated with age. The binary regression analysis showed that the AQP9 cut-off value had strongest impact on the development of caries with a hazard ratio of 2.79 (*p* = 0.009, Table 3) or periodontitis with a hazard ratio of 3.7 (*p* = 0.023, Table 4). However, when age is included in the binary regression analysis, the AQP9 cut-off remains the strongest factor with a hazard ratio of approximately 2 to 3, but it stays significant only for periodontitis (*p* = 0.024; supplementary Table 2) and not for caries (*p* = 0.068; supplementary Table 1).

**Table 3:** Binary logistic regression model for the occurrence of caries.

	*p*-value	Hazard ratio	95.0% CI	
			Lower	Upper
AQP9 cut-off	0.009	2.79	1.29	6.00
Gender male	0.801	0.907	0.423	1.942
Arterial hypertonia (yes)	*0.868*	0.916	0.325	2.581
Heart disease	0.477	1.613	0.432	6.015
Smoking (yes)	*0.224*	0.370	0.074	1.840
Years of smoking	0.067	1.063	0.996	1.134
Diabetes	1.000	0.000	0.000	

Significant values are in [italics].

**Table 4:** Binary logistic regression model for the occurrence of periodontitis.

	*p*-value	Hazard ratio	95.0% CI	
			Lower	Upper
AQP9 cut-off	0.023	3.709	1.195	11.506
Gender male	0.122	2.494	0.784	7.937
Arterial hypertonia (yes)	*0.079*	3.038	0.879	10.503
Heart disease	0.441	1,832	0.393	8.537
Smoking (yes)	*0.449*	1.965	0.341	11.310
Years of smoking	1.00	> 5	0.000	1.134
Diabetes	0.128	7.094	0.570	88.324

Significant values are in [italics].

**Fig. 4:**
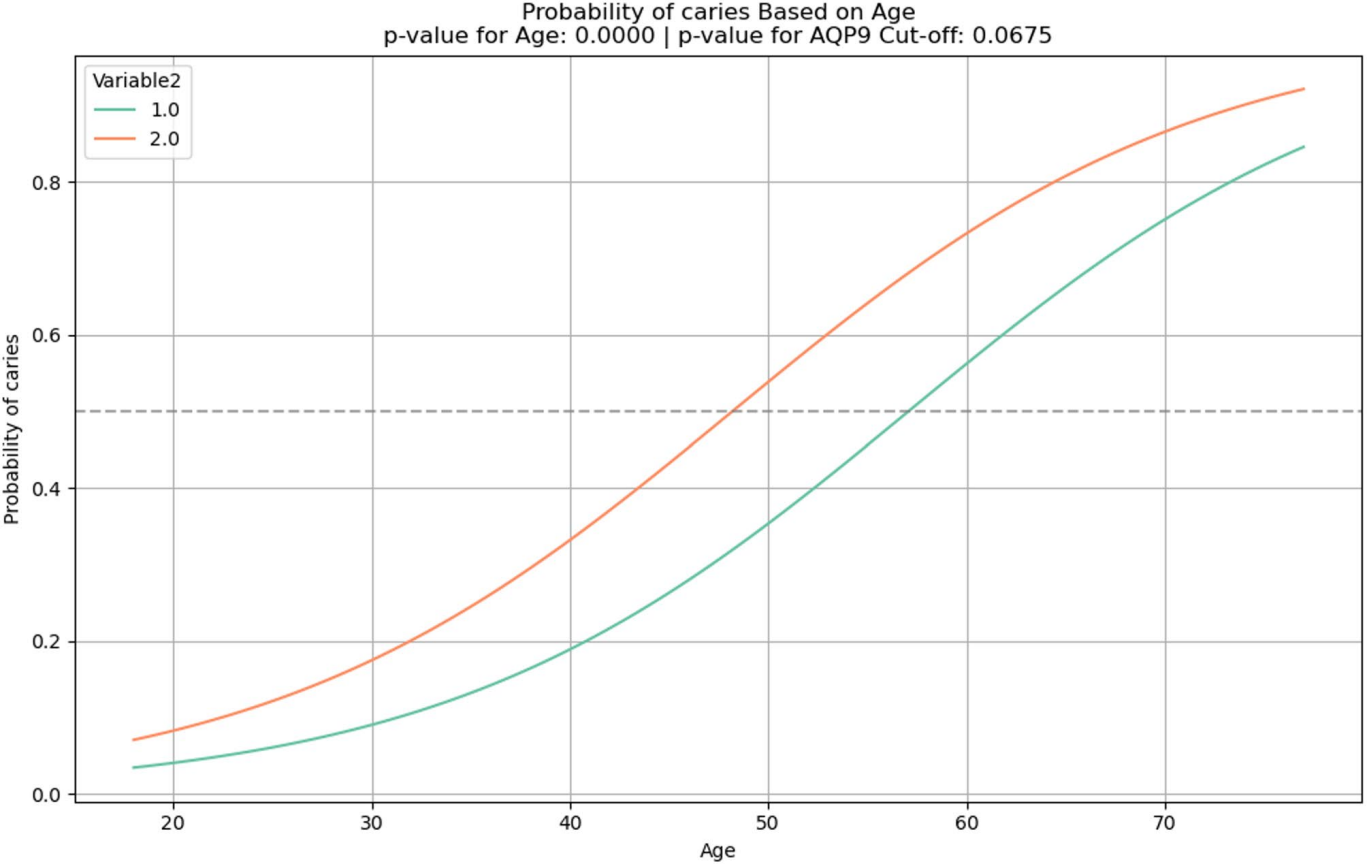
line plot for the probability of caries depending on age and stratified by the AQP9 cut-off value (Variable 2) 1 = below cut-off, 2 = above cut-off.

**Fig. 5:**
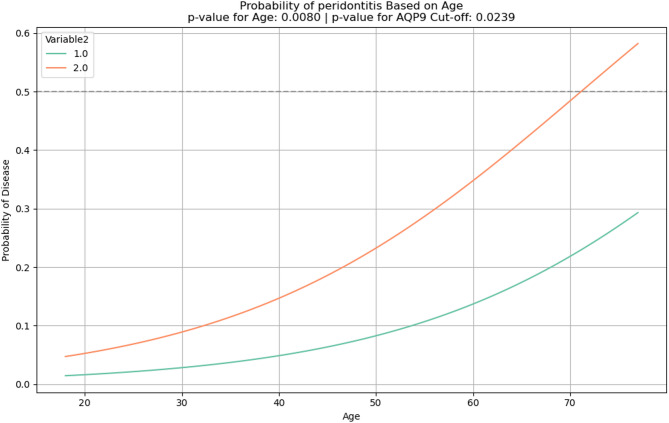
line plot for the probability of periodontitis depending on age and stratified by the AQP9 cut-off value (Variable 2) 1 = below cut-off, 2 = above cut-off.

As the occurrence of caries and periodontitis were correlated with age, a line plot was created to visualize the relationship between age and disease probability. The line plot shows how the probability of caries or periodontitis changes with age, separated by the categories of the AQP9 cut-off values. In both diseases the exceeding of the cut-off associated with a higher probability of developing caries (*p* = 0.0675, Fig. 4) or periodontitis (*p* = 0.0239, Fig. 5) but only in periodontitis it reached significance level.

## Discussion

Our study found that salivary AQP9 expression was higher in individuals with increased caries burden, as defined by DMFT-based categories, and in patients with periodontitis. In addition, AQP9 seems to be a risk factor in caries and periodontitis, with age being another important factor. Although the association between AQP9 expression and higher caries burden did not reach statistical significance in age-stratified models (*p* = 0.0675), the trend observed is consistent with the biological role of AQP9 and supports the need for further investigation. However, multivariate regression models and line plots indicate that the association between AQP9 and periodontitis is particularly strong and independent of age. With AUC values of approximately 0.63, AQP9 expression alone does not provide sufficient diagnostic accuracy for clinical application. However, as a biologically plausible and easily measurable salivary marker, AQP9 may contribute to a multi-marker panel for higher caries burden or periodontitis risk stratification.

To our knowledge, this is the first study to investigate the role of AQP9 in caries burden or periodontitis. Another study showed that AQP9 expression is increased in periodontitis cases compared to control cases^15^. An earlier study found that AQP9 is significantly upregulated in peri-implant soft tissue, suggesting its potential role in altered cell-ECM interactions and peri-implant tissue susceptibility^16^.

Evidence on acquired risk factors for caries, primarily from studies in children, highlights hyposalivation, smoking, and certain medical conditions as contributors. Hyposalivation (e.g., due to medications, radiation or Sjögren’s syndrome), maternal smoking, and poorly controlled type 1 diabetes are linked to increased caries risk, though the level of evidence supporting these associations is generally low^17^. Evidence for acquired risk factors for periodontal diseases in adults includes cardio-metabolic disorders, rheumatic diseases, hormonal changes, medications, and tobacco use. High-certainty evidence links poorly controlled diabetes and tobacco use to increased risk, while obesity, hormonal changes, and medications reducing salivary flow or causing gingival overgrowth show moderate to low certainty of association^17^.

We should discuss potential similarities between AQP9 and AQP5, including regulation of salivary flow and fluoride transport, and the age-related expression patterns observed in salivary glands.

Interestingly, AQP9 expression in our study exhibited age dependence, which had already been demonstrated for AQP5 in previous studies. Immunostaining showed strong AQP5 presence in juvenile mice, while Western blot analysis indicated an age-related decline in AQP5 protein levels, despite stable gene expression. Notably, AQP5 gene expression remained high in submandibular glands of both sexes, increased in parotid glands of older females, and was elevated in sublingual glands of young males, suggesting potential sex-related differences in AQP5 regulation^18^.

The exact role of AQP9 in caries and periodontitis remains speculative and no causal inference can be made. AQP9 might be linked to hyposalivation, which could serve as a potential mechanism increased number of affected teeth, as reduced salivary flow significantly impairs oral clearance^19^. However, AQP9 seems to be expressed in salivary glands, but there is not much known about the expression and function of AQP9 in them^20^. It is unclear which salivary glands exhibit high or low AQP9 expression^20^. As autoantibodies against aquaporins, particularly AQP8 and AQP9, are common in Sjögren’s syndrome (SS) patients and may contribute to salivary gland dysfunction, highlighting the need for further research on their pathogenic role^21^. Notably, these autoantibodies target the extracellular domains of AQP proteins and may directly interfere with their physiological function. In particular, anti-AQP-positive patients exhibited more severe xerophthalmia, pointing to a possible pathogenic role of these antibodies in exocrine gland dysfunction. The identification of AQP9 as a target of autoantibodies in SS is especially relevant to our findings, as it suggests that AQP9 dysfunction—whether through genetic regulation or immune-mediated mechanisms—may contribute to salivary gland impairment and hyposalivation, both of which are major risk factors for dental caries and periodontitis. This supports the hypothesis that altered AQP9 activity in saliva could serve as a biomarker of oral disease risk, potentially mediated not only by transcriptional regulation but also by post-translational immune interference.

Further studies are warranted to investigate the prevalence and functional effects of anti-AQP9 antibodies in patients with oral inflammatory diseases beyond SS and to explore their utility in diagnostic or therapeutic strategies.

Additionally, AQP9 similar to AQP5, could potentially influence fluoride levels in saliva, thereby affecting remineralization processes^22^.

We can discuss about AQP9’s potential involvement in salivary glycerol transport, influencing metabolic activity of cariogenic bacteria (e.g., *Streptococcus mutans*) and contributing to acid production and enamel demineralization.

AQP9 is able to transport glycerol^7^. Thus, it could regulate glycerol levels in saliva, which could influence the metabolic activity of cariogenic bacteria such as *Streptococcus mutans*^23^. Glycerol is a substrate that can be metabolized by these bacteria and contributes to the production of acids that demineralize tooth enamel^24^. Therefore, increased AQP9 expression may exacerbate sugar-induced dental damage.

Furthermore AQP9’s role in neutrophil function and local immune responses should be discussed. Recent studies suggest that neutrophils may degrade dentin and restorative materials in the gingival sulcus, possibly linking immune activity to caries development. Hence, the role of AQP9 in immune regulation may be critical for defense against caries-associated pathogens^11^. AQP9 is primarily found in neutrophils^11,13^. A recent study showed that the number of neutrophils in blood samples was higher in patients with severe periapical periodontitis than in those with mild pathology^25^. Historically, neutrophils were not thought to play a role in the pathogenesis of caries. Cell culture experiments suggest that both bacteria and neutrophils could have, among other things, direct degradative activity against both dentin collagen type 1 and/or methacrylate resin-based restoratives and adhesives, the most commonly used dental restoratives. Neutrophils are abundant leukocytes in the gingival sulcus, where they could easily reach adjacent roots or gingival and cervical restorations and could exert their degradative activity^26^. Thus, by modulating inflammatory responses in the oral cavity, AQP9 may influence the balance between protective and pathogenic factors in dental biofilms^24,26^. Understanding these mechanisms may open new avenues for preventive and therapeutic strategies targeting AQP9 in saliva.

Limitations of our study should be mentioned. A limitation of this study is that caries severity was defined based on the number of affected teeth (DMFT-based categories) rather than on lesion depth, activity, or surface-level characteristics; therefore, the findings reflect overall caries burden rather than the biological severity or progression of individual lesions. Further this study utilized qPCR to study AQP9 mRNA expression in saliva. A key limitation of this study is its observational, cross-sectional design, which involved the collection of biosamples and questionnaire data at a single time point. This approach precludes the ability to establish causal relationships between RNA expression levels and the presence of caries or periodontitis, limiting the findings to associations only. In further analyses it would be helpful to use immunohistochemistry or Western Blot to detect AQP9 protein expression in salivary glands and saliva samples. In addition functional assays to test water and glycerol permeability of AQP9 in vitro could be of interest^27^. Hence, the study is limited by the lack of protein-level validation (e.g., Western blot, IHC) and functional assays to confirm AQP9 activity in saliva. This limits the mechanistic interpretation of our findings. Future studies could also focus on developing specific AQP9 inhibitors to evaluate their effects on salivary composition and function^28,29^. In addition, smoking and the use of xerostomia-inducing medications were not part of the exclusion criteria and may have acted as confounding factors influencing salivary AQP9 expression. However, to minimize confounding, known systemic risk factors such as diabetes, cardiovascular disease, and smoking were documented and included in multivariable models. AQP9 expression was analyzed in relation to these variables, and no significant associations were found.

Although AQP9 has not been extensively characterized in salivary glands, its expression in immune cells such as neutrophils and its known ability to transport glycerol may contribute to local immunometabolic responses relevant to caries and periodontitis. Autoantibodies against AQP9 have been described in patients with Sjögren’s syndrome, suggesting a role in exocrine gland dysfunction. However, given its regulation by age and inflammation, we acknowledge that AQP9 may reflect a combination of primary and secondary disease mechanisms.

## Conclusion

This study demonstrates that increased AQP9 mRNA in saliva is associated with the presence of severe caries and periodontitis. While not sufficient as a standalone diagnostic tool, AQP9 may represent a useful biomarker within a broader panel. Further research is needed to validate these findings and explore the mechanistic role of AQP9 in oral diseases.

## Supplementary Information

Below is the link to the electronic supplementary material.

Supplementary Material 1

Supplementary Material 2

## Data Availability

The data that support the findings of this study are not openly available due to reasons of sensitivity and are available from the corresponding author upon reasonable request.

## Declarations

### Trial registration

The trial was registered in the German Clinical Trial Registry (DRKS), the trial registration number is DRKS00032425 and the date of registration was August 16, 2023.
