# Supplementary material for: Salivary AQP9 mRNA expression is associated with caries and periodontitis prevalence
Source: Sci Rep. 2026 Feb 13;16:6507. doi: 10.1038/s41598-026-37980-3 (PMC12909861; doi:10.1038/s41598-026-37980-3)
Supplement: Supplementary file 1 — Supplementary Material 1 [file 41598_2026_37980_MOESM1_ESM.docx]

Supplementary table 1: binary logistic regression model for the occurrence of caries

|  | **p-value** | **Hazard ratio** | **95.0% CI** | |
| --- | --- | --- | --- | --- |
|  |  |  | **lower** | **upper** |
| **AQP9 cut-off** | **0.068** | **2.12** | **0.95** | **4.8** |
| **Age** | <0.001 | 1.09 | 1.05 | 1.13 |

Supplementary table 2: binary logistic regression model for the occurrence of periodontitis

|  | **p-value** | **Hazard ratio** | **95.0% CI** | |
| --- | --- | --- | --- | --- |
|  |  |  | **lower** | **upper** |
| **AQP9 cut-off** | **0.024** | **3.357** | **1.117** | **9.602** |
| **Age** | 0.008 | 1.058 | 1.174 | 1.103 |
