# Supplementary figures and images for: Salivary AQP9 mRNA expression is associated with caries and periodontitis prevalence
Source: Sci Rep. 2026 Feb 13;16:6507. doi: 10.1038/s41598-026-37980-3 (PMC12909861; doi:10.1038/s41598-026-37980-3)

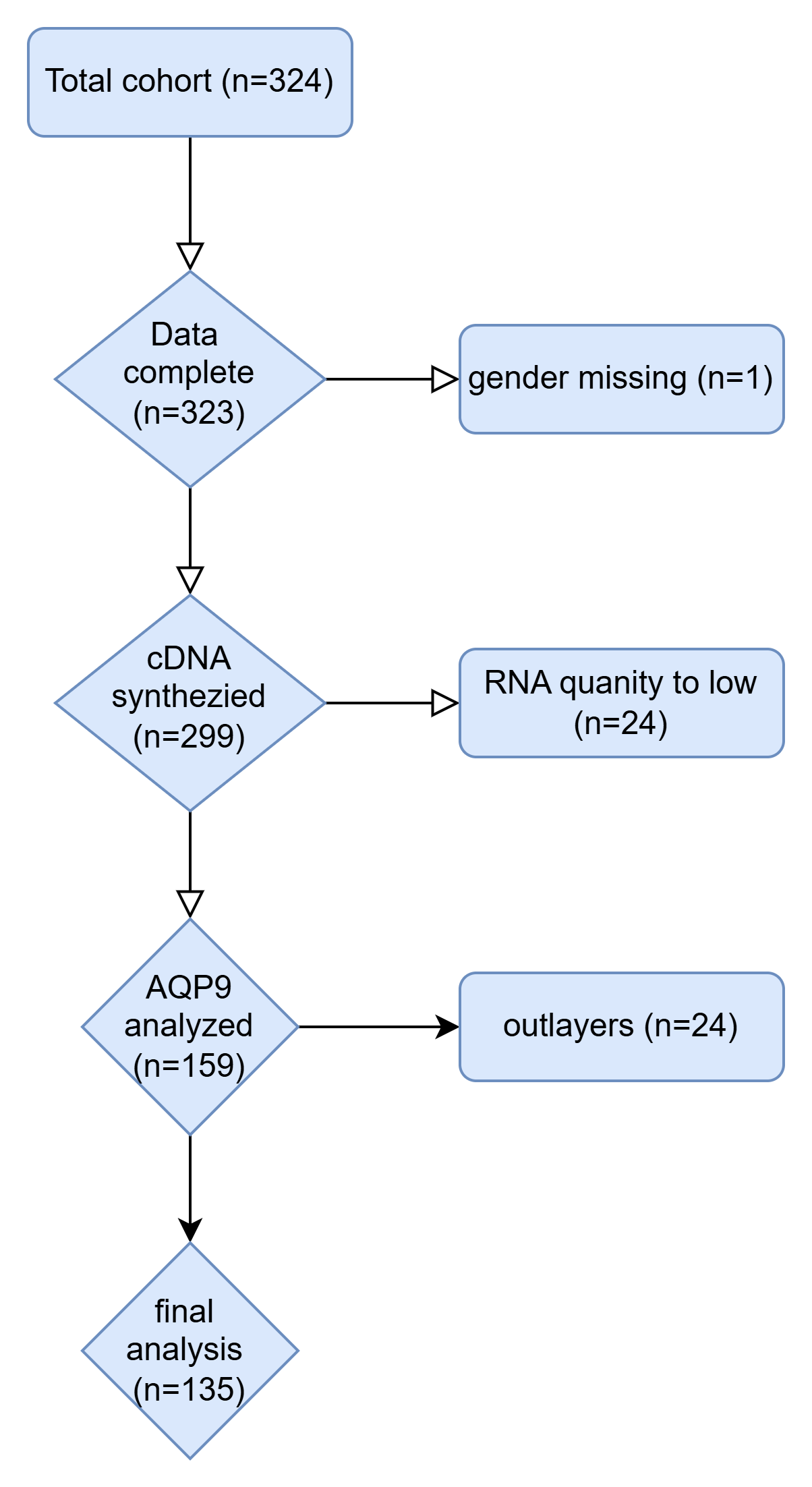


Suplementary Figure 1: flow diagram oft he study cohort

Supplement: Supplementary file 2 — Supplementary Material 2 [file 41598_2026_37980_MOESM2_ESM.docx]
